# Observation: unlocking, assessing, and nurturing creative problem solving

**DOI:** 10.3389/fpsyg.2025.1540501

**Published:** 2025-06-02

**Authors:** C. June Maker, Kadir Bahar

**Affiliations:** ^1^Department of Disability and Psychoeducational Studies, University of Arizona, Tucson, AZ, United States; ^2^Department of Educational Psychology, University of Georgia, Athens, GA, United States

**Keywords:** observation, creative problem solving, discover, performance-based assessments, creativity in behaviors, talents, play-based assessments, teaching for creativity

## Abstract

The purpose of this article was to advocate and provide methods for observation of behavior as a method for assessing and developing creativity during problem solving. To accomplish this purpose, the authors (a) outlined the advantages of observing people’s actions as a method for unlocking, assessing, and nurturing creative problem solving; (b) explained briefly the conceptual framework for the research underlying these recommendations; (c) presented results of research on observable behaviors that are indicators of abilities in creative problem solving in diverse domains of talent; and (d) described activities, experiences, and materials that have been used to unlock, assess, and nurture creative problem-solving capability and skills. Research on the Discovering Intellectual Skills and capability while Observing Varied Ethnic Responses (DISCOVER) in which performance-and play-based assessments of children and young adults (ages 3 to adult) were designed, field-tested, and validated over a 37-year period were the basis for making recommendations for behaviors to observe and methods for eliciting them. Research has been conducted in several countries (e.g., Australia, Bahrain, Canada, Chile, France, Hong Kong, Lebanon, Mexico, Taiwan, Thailand, and United Arab Emirates) and languages (e.g., Arabic, Chinese, English, French, Navajo, Spanish, Thai, and Taiwanese).

## Introduction

Assessments of creativity include tests of divergent thinking, attitudes toward creativity, and self-assessments of characteristics and attitudes related to creativity. Most involve written or written and graphic formats for responses [e.g., Torrance Tests of Creative Thinking (TTCT; Torrance, 1966); Test of Creative Thinking—Drawing Production (TCT—DP; Urban, 2005)]. Recently, online versions have been designed [e.g., the Evaluation of Creative Potential (EPoC; Lubart et al., 2013; Lubart et al., 2019)]. An often-overlooked format is observation of actual performance in situations in which creative actions are elicited. A leader in creativity assessment, Torrance (1973) published a checklist of 18 categories of observable behaviors he describes as “non-test indicators of creative positives.” The behaviors are in categories such as “ability to express feelings and emotions,” “articulateness in role-playing and storytelling,” “enjoyment and ability in visual art,” “fluency and flexibility in non-verbal media,” “enjoyment and skills in small-group activities, problem solving, etc.,” and “originality of ideas in problem solving” (pp. 5–8). Later, as an alternative to written assessments for young children, Torrance (1981) designed Thinking Creatively in Action and Movement (TCAM). Following his lead, Maker and colleagues (Alfaiz et al., 2020; Bahar and Maker, 2020; Maker, 1993, 1994, 1996, 2001, 2005, 2020a, 2020b, 2021; Maker et al., 1994, 1996, 2023a; Zimmerman et al., 2020) designed a series of performance-based assessments: Discovering Intellectual Strengths and Capabilities while Observing Varied Ethnic Responses (DISCOVER). However, our approach was different, in that we did not list the behaviors to observe when creating assessments of particular domains and age groups. We asked observers from varied backgrounds who were trained in administering the tasks to tell which students exhibited superior problem solving behaviors, which included creativity in the open ended problems. In the research section, this method is described more fully.

In this article, the intention is not to review the many different ways to assess creative problem solving, but to advocate and describe assessments involving observation of behavior. Although other methods can be effective and can be combined to provide a more complete picture of an individual’s abilities, what people *do* often is a better indicator of their capabilities than what they *say*. One way to think about this is that when responses are written, a person’s creative abilities are filtered through their linguistic capability; and when responses are drawn, their creative abilities are filtered through their visual/spatial capabilities. Observations also are filtered: through the eyes and thinking of the observer. Both observation and writing or drawing complement each other.

## Conceptual framework and design elements

Amabile’s (1996, 2013) theory and research was the conceptual basis for designing the DISCOVER assessments and the research of Getzels and Csikszentmihalyi (1967, 1976) provided the structure for designing tasks (Maker, 2021). In Amabile’s theory, creativity consists of *domain general* and *domain specific* knowledge, skills, and abilities; *creativity relevant* processes, skills, and abilities; and *motivation*. Building and elaborating on her theory, Lubart et al. (2019) identified ingredients that contribute to “creative potential” or the potential for creating: *cognitive* traits such as knowledge and flexibility, *conative* traits such as personality characteristics, and *motivation*, emotions and other affect-related traits, and *environmental* characteristics. A person’s potential for demonstrating creativity depends on the fit between her or his characteristics and the requirements of the task.

Our structure for designing tasks was based on the continuum developed and implemented in the research of Getzels and Csikszentmihalyi (1967, 1976) in their studies of creativity in science and art. In their approach, different types of problems or tasks were presented to individuals on a continuum from closed to open. The continuum included three types based on how much information was given about the task. In the research on DISCOVER (Maker, 1993, 2005, 2021), Getzels and Csikszentmihalyi’s continuum was expanded to include six types to enable observers and participants to make a transition from closed to open. In *closed* tasks (Types I and II), the problem is well-defined, the method is specified, and only one right answer is accepted (e.g., make this shape with these materials. Show the shape and provide the materials. Expect an exact replica.). In a *semi-open* task (Types III and IV), the problem is well-defined, one or a range of methods is possible, and a range of answers or solutions is accepted (e.g., make an animal with these materials. Expect the construction to be an animal, but different animals and different elements of animals are accepted.). In an *open* task (Types V and VI), the problem is defined for Type V, but neither the problem, method, or solution is specified for Type VI (e.g., make anything you want to make.). Materials are available, but participants can use other materials if they want to do so.

**Figure 1:**
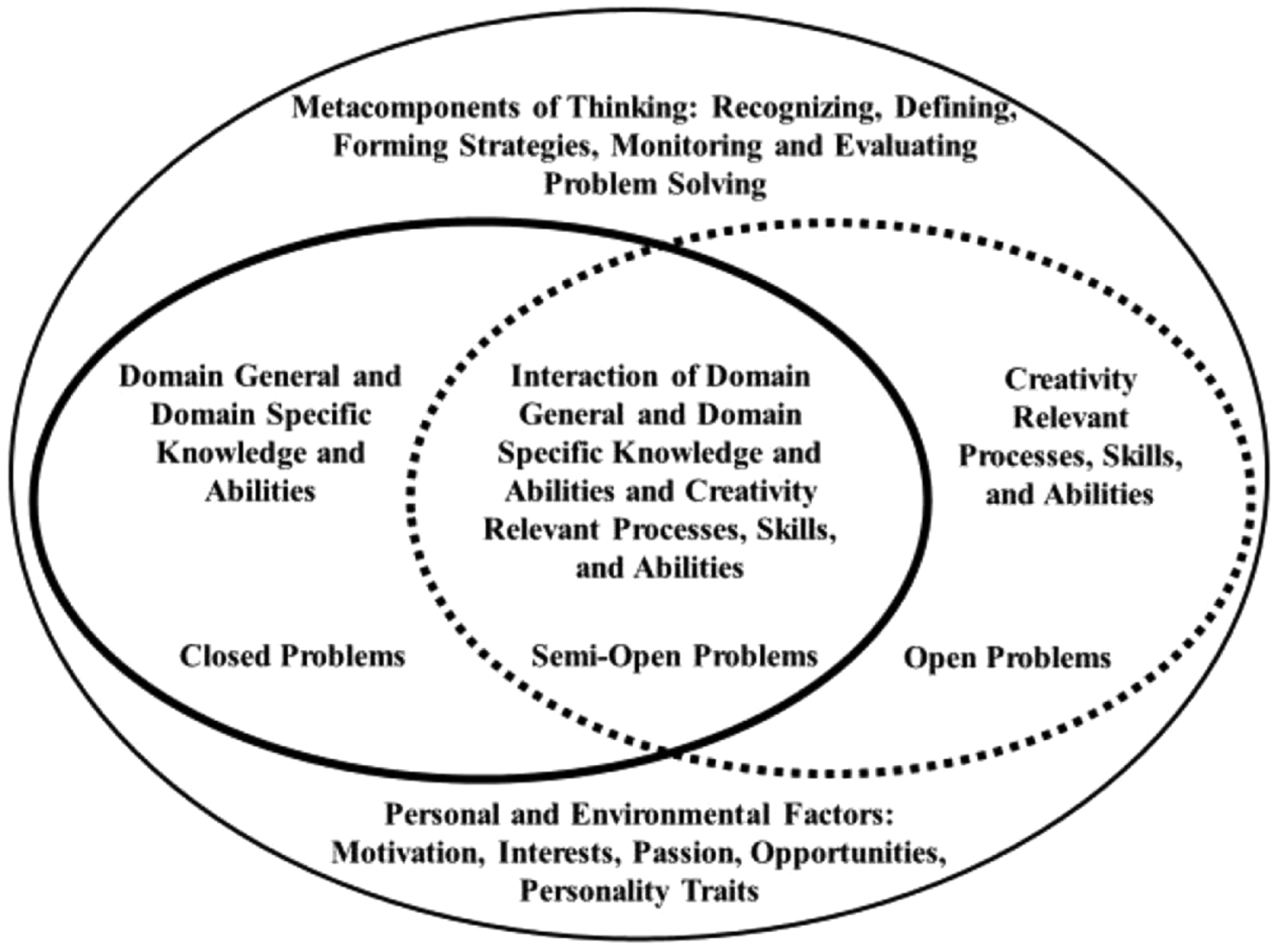
Integration of problem-solving components. Reprinted from Marker (2021).

Later, as a result of using the six types, the team decided to combine like types to make the assessments more practical. Figure 1 shows the connections among the elements of the conceptual framework and the structure of the tasks. *Closed* tasks require domain general and domain specific knowledge, skills, and abilities; *open* tasks require creativity relevant processes, skills, and abilities; and *semi-open* tasks require both. Motivation or desire to do the task is essential for all problem solving.

Because creativity contains both domain-specific and domain-general aspects (c.f., Amabile, 2013; Beaty et al., 2023; Kaufman et al., 2017; Steffens, 2022; Sternberg, 2005), an essential part of the design was a theory to guide decisions about the domains to be included. In the beginning, the DISCOVER assessments were based on Gardner’s (1983) theory of multiple intelligences in which he originally identified seven ability domains (bodily-kinesthetic, interpersonal, intrapersonal, linguistic, logical-mathematical, musical, spatial), and later added naturalistic ability (1999). Another important aspect of his theory was to define intelligence as problem solving in a way that included all the problem types in Getzels and Csikszentmihalyi’s (1967, 1976) structure. Intelligence is “…a set of skills of problem solving enabling the individual to resolve genuine problems or difficulties…and … the potential for finding or creating problems—thereby laying the ground work for the acquisition of new knowledge” (Gardner, 1983, p. 60). This definition provides a rationale for integrating, rather than separating, creativity and intelligence (Maker, 1993, 2021). Creativity also has been defined as problem solving: “…the process of sensing gaps or disturbing, missing elements; forming ideas or hypotheses concerning them; testing these hypotheses; and communicating the results, possibly modifying and retesting the hypotheses” (Torrance, 1962, p. 21).

As a result of implementing the DISCOVER assessments and conducting research on their validity, the talent areas (e.g., domains of ability) have been re-defined (Maker and Anuruthwong, 2003; Maker, 2021). Ten talent areas have been identified and described. Many are similar to the ability domains identified by Gardner (1983, 1999), but are expansions and divisions as outlined by Maker et al. (2021): auditory, bodily/somatic, emotional/intrapersonal, linguistic, mathematical, mechanical/technical, moral/ethical/spiritual, scientific/naturalistic, social/interpersonal, and visual/spatial. In our most recent research with young children, to enable observers to discuss their perceptions of participants’ behaviors with other observers, ability domains have been placed into three groups: *Arts* (auditory, bodily/somatic, visual/spatial); *Academic and Science, Technology, Engineering, and Math (STEM)* (linguistic, mathematical, mechanical/technical, scientific/naturalistic); and *Leadership* (emotional/intrapersonal, moral/ethical/spiritual, social/interpersonal) (Maker et al., 2023a).

The reliability and validity of the DISCOVER assessments have been documented in several studies. For example, in one study, the DISCOVER mathematics assessment, administered at the beginning of the year, explained 20% of the variance in Stanford-9 math scores at the end of the year, providing strong evidence for predictive validity (Sak and Maker, 2005). In two studies, researchers documented how content validity was established using a comprehensive review by teachers, specialists, and researchers (Bahar and Maker, 2011; Bahar and Maker, 2020). In other studies, the inter-rater reliability coefficients were investigated and found to be over 0.94 (Maker, 2005; Sarouphim, 1999; Griffiths, 1996). For a list of investigations of the validity and reliability of the assessments and behavior checklists, readers may consult the resources list in Supplementary File 1. Also important is that these instruments were translated into other languages and used internationally over the years. Consequently, multiple researchers have studied the implementation and research on these assessments in different countries (including validity and reliability of each assessment and behavior checklist). We also listed some of these studies in the resources section (see “Implementation and Research on Assessments in Different Countries” in the resources section).

## Principles and methods for designing the DISCOVER performance and play-based assessments

**Figure 2:**
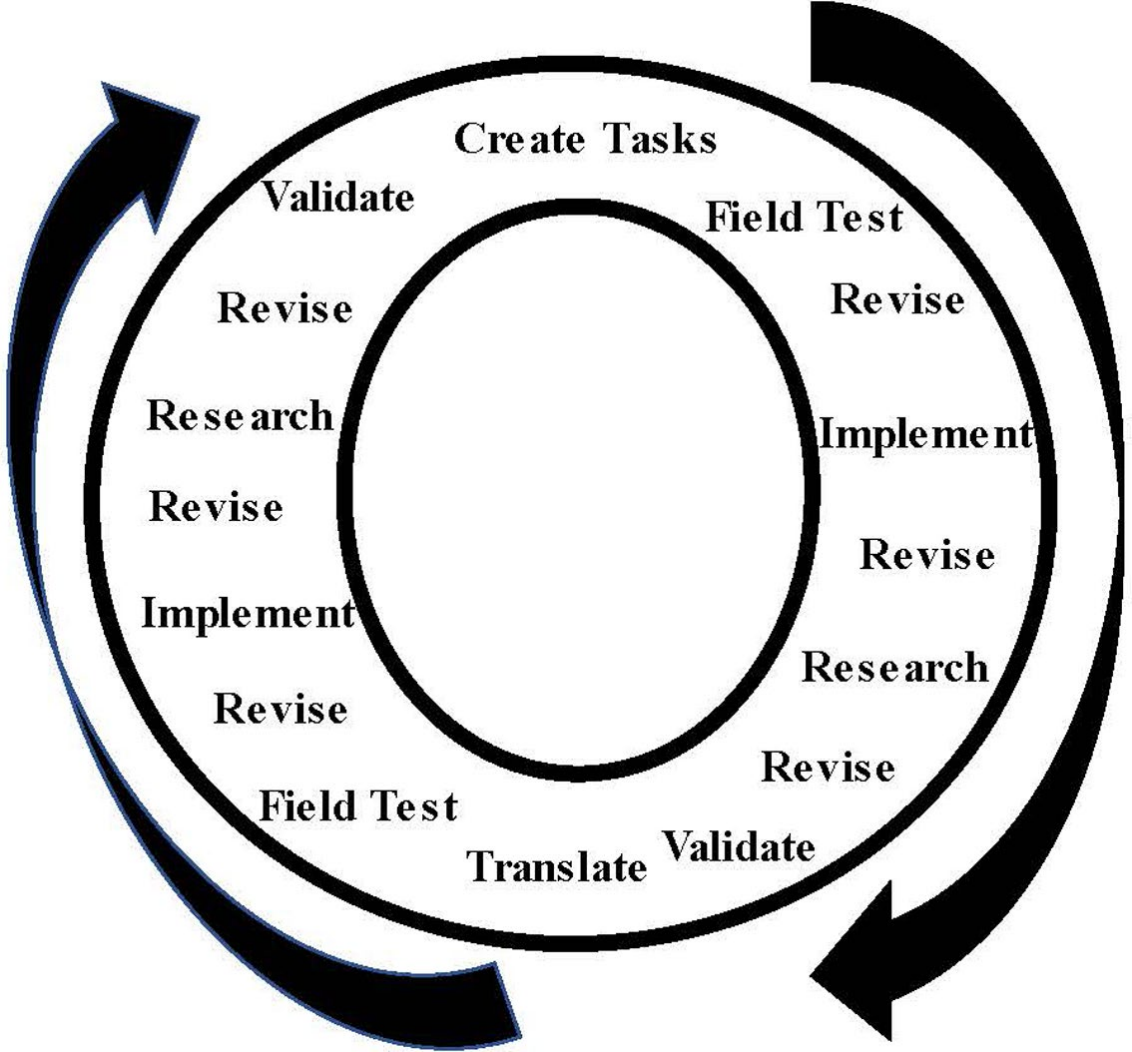
Field-testing and revision process for DISCOVER assessments. Reprinted from Maker et al. (2023a).

Within the conceptual framework and structure for designing the assessments, certain principles were followed: (a) tasks needed to be developmentally appropriate and engaging to individuals of the age group being assessed; (b) materials needed to be developmentally appropriate, durable, flexible, and used to create a variety of products; and (c) tasks needed to have the potential to elicit creative problem-solving behaviors in the domain of assessment. Talent development principles and classroom experiences were important aspects of the design of tasks (Maker and Schiever, 2010; Maker and Pease, 2021). Tasks and questions to stimulate participation were chosen to simulate a setting similar to a classroom or program for talent development, including special programs for gifted and talented students. They were designed to “unlock” talents by engaging children and youth in interesting activities. Observers also asked provocative questions to stimulate creative thinking and actions related to the domain of assessment. Tasks and materials were field-tested in a variety of settings with children, youth, and adults using a process alternating between assessment of individuals or groups, revision, assessment of different groups or individuals of the same age, research, translation, revision, and research. The process lasted as long as necessary for developers and observers to agree on the final form (Maker et al., 2023a). Figure 2 shows this process, including field-testing and revision after translation into another language.

Although the researchers followed certain principles when designing the DISCOVER assessments, each of these tools had unique assessment methodology and scoring methods. For example, a criterion-referenced scoring method was used to evaluate creative behaviors in mathematical problem solving, while a sample-based scoring method was used in the life science assessment. A list of the studies providing descriptions of assessment methodologies used in each assessment tool has been provided in the resources section (Supplementary File 1).

### Research methods to identify behaviors to observe

An essential aspect of this process in the context of promoting observation as an important way to unlock, assess, and nurture creative problem solving is the validity of the behaviors to observe that indicate strengths and talents in the domains being assessed. These indicators have been identified and validated through the same process as the tasks. Important elements of the process included (a) having observers from the culture and language of those being assessed; (b) including observers of different ages and varied backgrounds such as teachers, parents, administrators, researchers, artists, performers, and others; and (c) identification of *observable* behaviors, not *inferences* about abilities made by observers. For this last element, when a new assessment was being field-tested, no behaviors were listed for observers. After an activity was completed, observers met to discuss the performance of participants. First, developers and researchers asked observers to tell which participants were “superior problem solvers” in the activity. When they described a particular individual, they were asked “What did he/she do or say that led you to believe he/she is a superior problem solver?” If they made an inference such as “highly motivated,” they were asked to identify specific, observable behaviors or statements made by the individual “What did he/she do or say that led you to believe he/she is highly motivated?” Answers such as “did not want to quit even when others had finished,” “showed involvement in task (focused on own work rather than that of others, not easily distracted)” and “worked continuously” were the kinds of statements included on observation checklists (Maker, 2005; Rogers, 1998).

All decisions about behaviors to include on checklists were made by consensus of experts and observers. When making final decisions about ratings to assign for a particular individual’s performance, all observers in a classroom or group of students met to view video and audio recordings, photos of constructions, and notes made by observers. Together, they decided on the ratings for all individuals in the classroom or group. This process is similar to the Consensual Assessment Technique (CAT) designed by Amabile (1982) and used in creativity assessments. However, unlike CAT, observers meet and reach consensus rather than submitting their results to researchers, who then compile them.

Space does not permit inclusion of results of research or descriptions of all 10 assessments. At the end of the article, a short synthesis of research is available accompanied by a section of the reference list categorized according to research and practice related to the content of this article. These references are available in various publications for those wishing to have more information.

## Unlocking, assessing, and nurturing abilities: how and what to observe

In the following sections one assessment from the *Arts* and *Leadership* groups and two assessments from the *Academic and STEM* group are described. Each section includes the materials to use, tasks for participants, questions to be asked by observers, and behaviors to observe that indicate creativity. Assessments have been designed for young children, approximately age 4 through 6, elementary school (approximately age 6 or 7 through 12), middle and high school (approximately age 13 through 18). The middle and high school assessments also are appropriate for adults. Only the open tasks and questions are included in this review rather than all three types (closed, semi-open, and open) because the open tasks and questions are essential for unlocking, assessing, and nurturing creativity. Closed and semi-open are important for assessing the full range of creative problem solving, including domain-related knowledge and skills, and also can serve as warm-up experiences leading to the open-ended tasks.

All behaviors listed are results of research in several studies, so specific references are not provided for each behavior.

### Academic: linguistic ability

#### Materials

**Figure 3:**
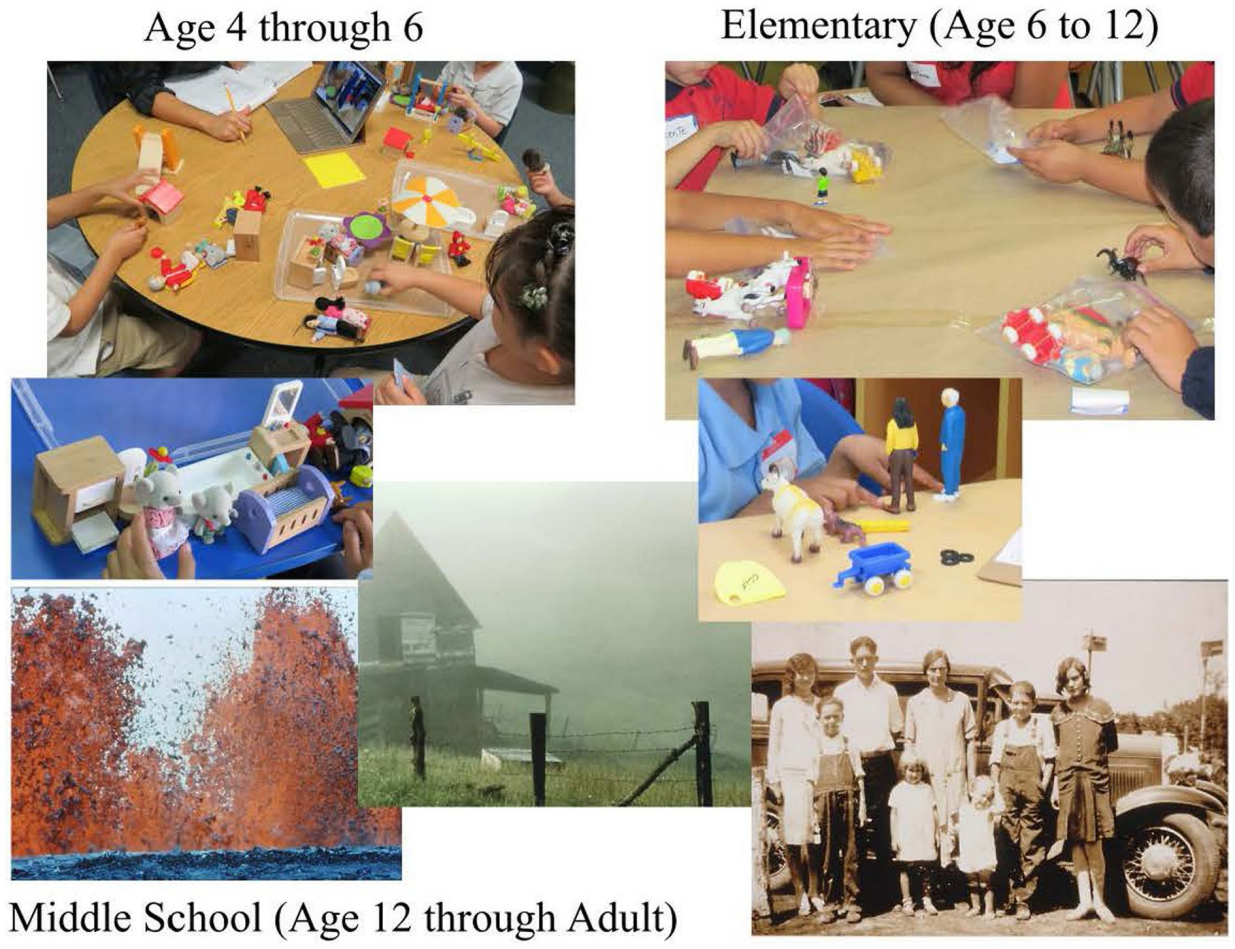
Materials for linguistic assessments for all levels.

Students in preschool (age 4) to the end of elementary school (approximately age 12) have bags or boxes of toys, including people, animals, furniture, and various things. Each box or bag is different, but all contain the same categories of items such as those listed above to enable consistency while preventing participants to copy from someone else. Students and young adults from middle school through high school and adult are shown slides with different environments. They also are given a small bag with items such as a shell, a coin, a rock or crystal, and any other items that are culturally appropriate. Figure 3 shows materials for all levels.

#### Open-ended tasks and questions to stimulate creativity

- *Preschool through elementary school*: Say “*Tell a story about any or all of your toys*. *You can tell any story you want to tell*.” If a child says very little, you can ask once “*What else happened?*” Children in Kindergarten and grade 1 can be given another task. Say “*Draw a picture that tells a story*.” After each child is finished, say “*Tell me about your picture*.” Record the response verbatim. Grades 2 through 6 can be given a slightly different task. Say “*Write about anything you want to write about*. *Spelling and punctuation do not matter*. *I am interested in your ideas or story*.”
- *Middle school through adult*: After showing the slides to all students in the group, paper copies of the pictures shown as slides are made available. Say “*Choose one of the pictures and imagine you are in the picture*. *Write or tell about your experience*. *You also can imagine you found one or more of the items in your bag when you were in the picture*.”

#### Observable behaviors that are indicators of linguistic creativity based on research

- Gives descriptions easily and fluently.
- Gives detailed descriptions.
- Uses more than one language.
- Chooses colorful or unusual adjectives and adverbs.
- Makes long sentences.
- Makes complex sentences (e.g., many words, many phrases, many parts of speech).
- Includes many details.
- Includes action.
- Includes feelings.
- Includes humor.
- Creates a visual image for the listener.
- Creates a story or other form with a plot or central idea.
- Creates a story or other form with many different themes.
- Includes dialogue or conversation.
- Changes voice to represent different characters.
- Creates an ending that is consistent with the story or structure (e.g., poem, news article, dialogue, or other form).
- Creates a surprise ending that is plausible.

#### Sample linguistic products that exhibit creativity

**Figure 4:**
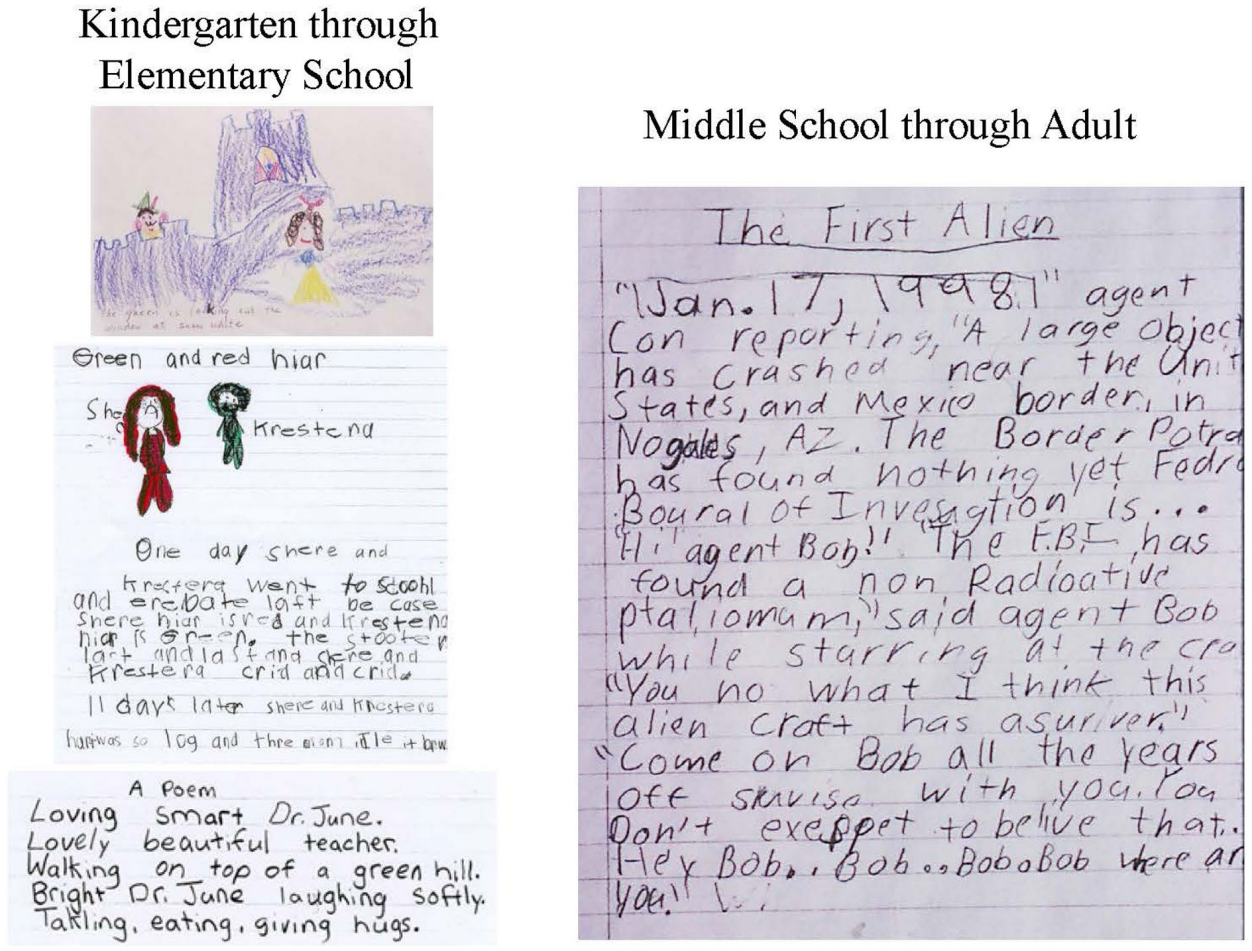
Examples of creative linguistic products.

When observing, processes used by participants can be observed. Products resulting from that process also can be evaluated. Verbal storytelling is audio recorded, then transcribed verbatim for evaluation. Both the process and the product are important. Figure 4 has examples of linguistic products that exhibit creativity.

### STEM: mechanical/technical ability

#### Materials

**Figure 5:**
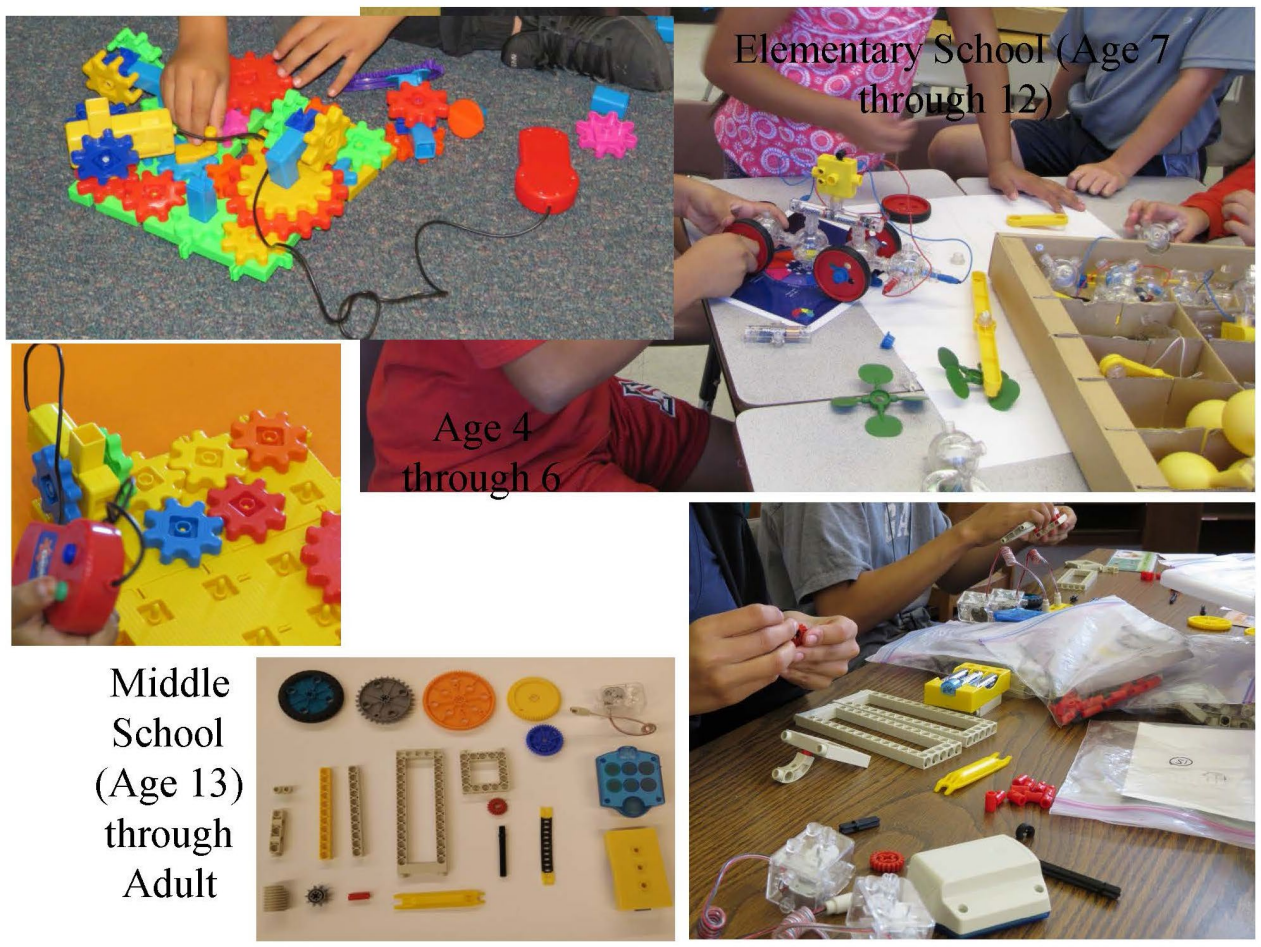
Materials for mechanical/technical assessment for all levels.

For all assessments of mechanical/technical ability, students are given boxes of materials that can be used to make mechanical constructions. All include batteries and connections for powering the constructions. All students in the group have the same number and type of items. Figure 5 shows materials for all levels. The most important principle to follow is to provide materials that can be used in many different ways, and to remove any instructions for making particular constructions.

#### Open-ended tasks and questions to stimulate creativity

- *Age 4 to 6*: The closed task is to make a simple gear train like the one demonstrated so they know what it is and how it works. For the open task, say “Make *your own gear train*.” After they have worked on their gear trains for 5 min or more, go to each child. Show the battery-operated gear and other pieces that can be used to make more complex gears. Show the pieces one at a time and ask: “*How can you use this in your gear train?*” If the child has already used the pieces, ask the him/her to demonstrate how the gear train works. At the end, make sure to ask all children to demonstrate their gear trains.
- *Elementary school through adult*: “*Make a machine that moves by using the remote and motors*. *Make something different from what you have already made*. *It needs to be your own design*.” When each individual is finished with his/her machine, ask the person to show how the machine works.

#### Observable behaviors based on research that are indicators of mechanical/technical creativity: age 4 to 6

- Uses the principles of energy transfer to construct gears.
- Adds many gears that function as part of the gear train.
- Makes horizontal and perpendicular gears that function.
- Uses gear pieces in a variety of ways.
- Makes a gear train different from the example.
- Makes a complex gear train with many pieces.
- Integrates medium and/or large gear that function as part of the gear train.
- Integrates chain gear that functions as part of the gear train.
- Integrates motorized gear that functions as part of the gear train.
- Integrates non-gear parts that function as part of the gear train.
- Changes nonworking gear parts to make a working gear train.
- Integrates platforms in a unique way as part of the gear train.

#### Observable behaviors based on research that are indicators of mechanical/technical creativity: elementary school through adult

- Is complex.
- Has many functions.
- Is stable.
- Is unique.
- Has 2 or more motors that function.
- May use only 1 motor, but has a unique design.
- Moves right and left or goes in circles.
- Has a design different from the vehicle the student made.
- Has gears powered by a motor or motors.
- Has gears with a function.
- Explanation of machine demonstrates understanding of gear ratio
- Has chains to connect gears or wheels.

#### Sample mechanical/technical products that exhibit creativity

**Figure 6:**
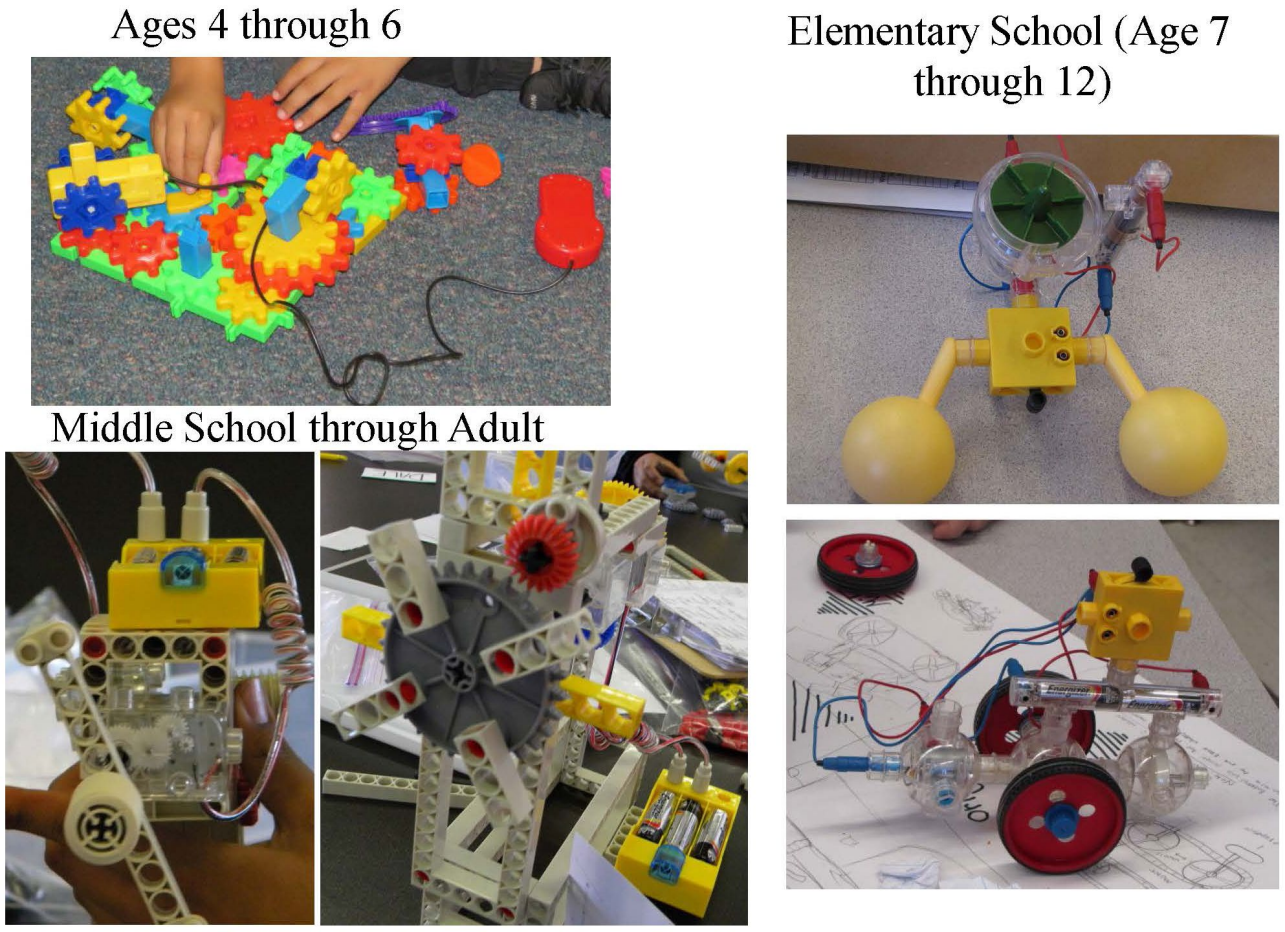
Examples of creative mechanical/technical products.

When observing, processes used by participants can be observed. Products resulting from that process also can be evaluated. Both are important. Figure 6 has examples of mechanical/technical products that exhibit creativity.

### Leadership: social/interpersonal ability

#### Materials

**Figure 7:**
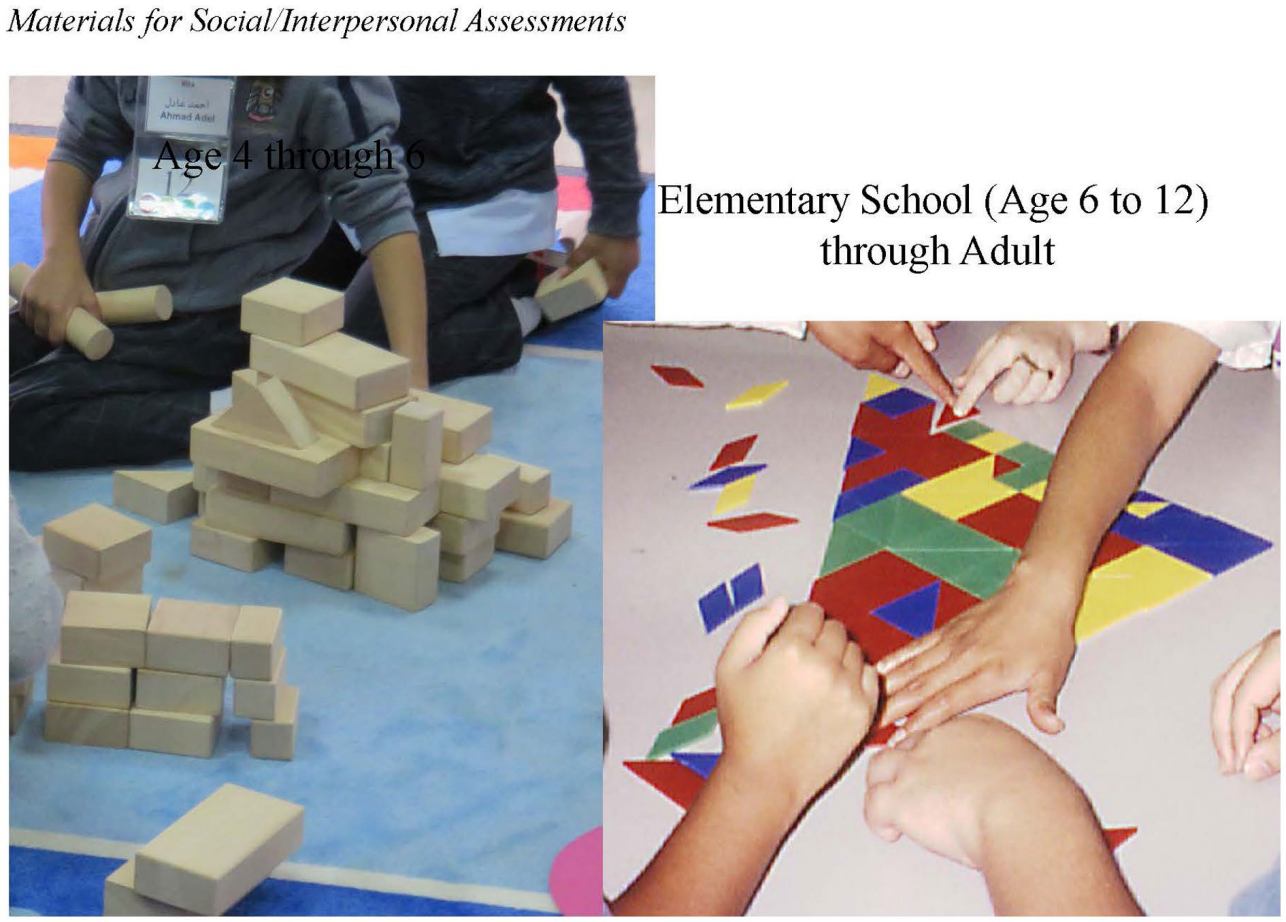
Materials for social/interpersonal assessments.

Students are placed in a group of four young children and a group of five in elementary through high school and adult. Each group is given a set of materials that can be used to construct what they are told to make. Figure 7 shows the materials we have given groups that are developmentally appropriate and useful for making a construction in which participants need to collaborate.

#### Open-ended tasks and questions to stimulate creativity

- *Age 4 to 6*: In this assessment, the only task is open-ended. Children in the group are seated in a circle with a pile of kindergarten blocks in the center. The observer says “*Make a bridge, all together*.” Children are shown a picture of a bridge made with the blocks, and it is taken away after the children have looked at it. The task is not to copy the picture, but to make their own bridge. After 5 min or so, if children are working separately or in pairs, stop the activity, put all blocks into the center again, and say “*Make ONE bridge, all TOGETHER*.” Make a motion that includes everyone. After the group has completed a bridge or the time limit for the activity is reached, interview each child. Ask “*What did you do to help the group?*”
- *Elementary school through adult*: In this assessment, the only task is open-ended. Give each participant a set of 21 Tangrams of different geometrical shapes; three triangles (6 large, 3 medium, and 6 small), (3 small parallelograms, and 3 small squares). Say “*Make one triangle with as many pieces as you can*.” If they start making individual triangles, remind them “*Make one triangle together, using as many pieces as you can*.” After the group completes the triangle, ask each individual questions such as “*How did you help or hinder the group?*” “*How did the group work together?*”

#### Observable behaviors based on research that are indicators of social/interpersonal ability

- Manages own emotion(s) in the group.
- Identifies and/or responds to emotion(s) shown by others.
- Manages own and/or group disappointment effectively.
- Shows interest in others in the group and/or observers.
- Shows kindness to others.
- Shows and encourages humor in a positive way.
- Cooperates with others verbally (e.g., agreeable with others’ ideas, uses “us,” “we,” “let us”).
- Cooperates with others in a nonverbal way (e.g., accepts the placing of an added piece).
- Listens to and responds to others.
- Others listen to and respond to him/her.
- Others follow his or her suggestions.
- Makes positive comments to others (e.g., says good things about what they are doing).
- Encourages others (e.g., tells them this is a good idea).
- Shows pleasure when others contribute (e.g., smiling, laughing, encouraging).
- Shows patience (e.g., waits for others rather than interrupting).
- Shows initiative in a positive way.
- Organizes others to complete the task.
- Gives help to others (gives away pieces, gives hints).
- Changes behavior if it seems to affect the group negatively.
- Observes that everyone is participating and no one is left out.
- Directs others in a positive way (talks with others, makes offers).
- Identifies own behavior.
- Tries to resolve a conflict.
- Finds ways to include others (offers a block or a Tangram piece).

### Sample social/interpersonal products that exhibit creativity

**Figure 8:**
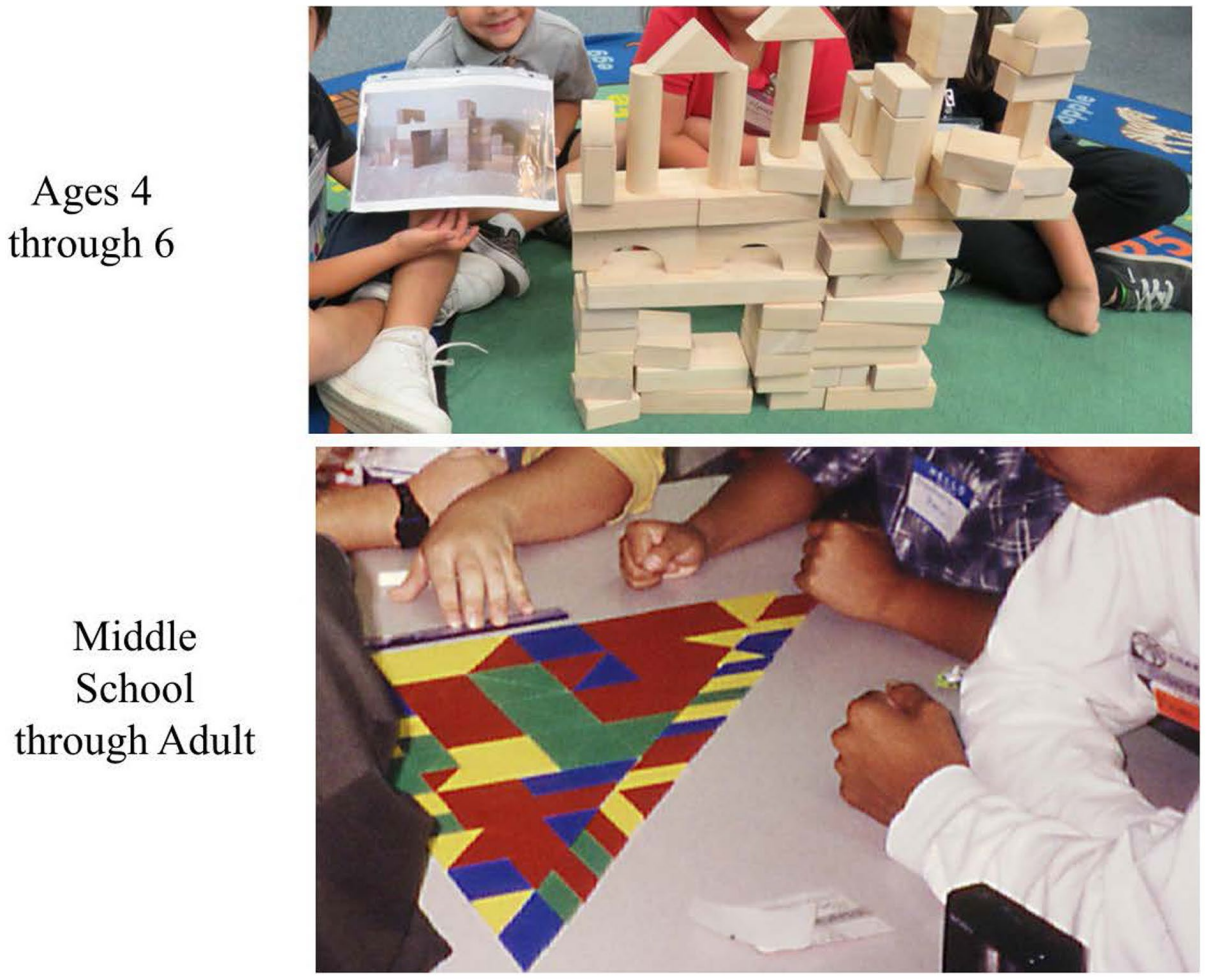
Examples of social/interpersonal products that show creativity.

When observing, the processes used by participants are the basis for making decisions. However, the products resulting from that process can provide clues to the level of collaboration in the group. Figure 8 has examples of social/interpersonal products that can be indicators of creativity and collaboration.

### Arts: visual/spatial ability

#### Materials

**Figure 9:**
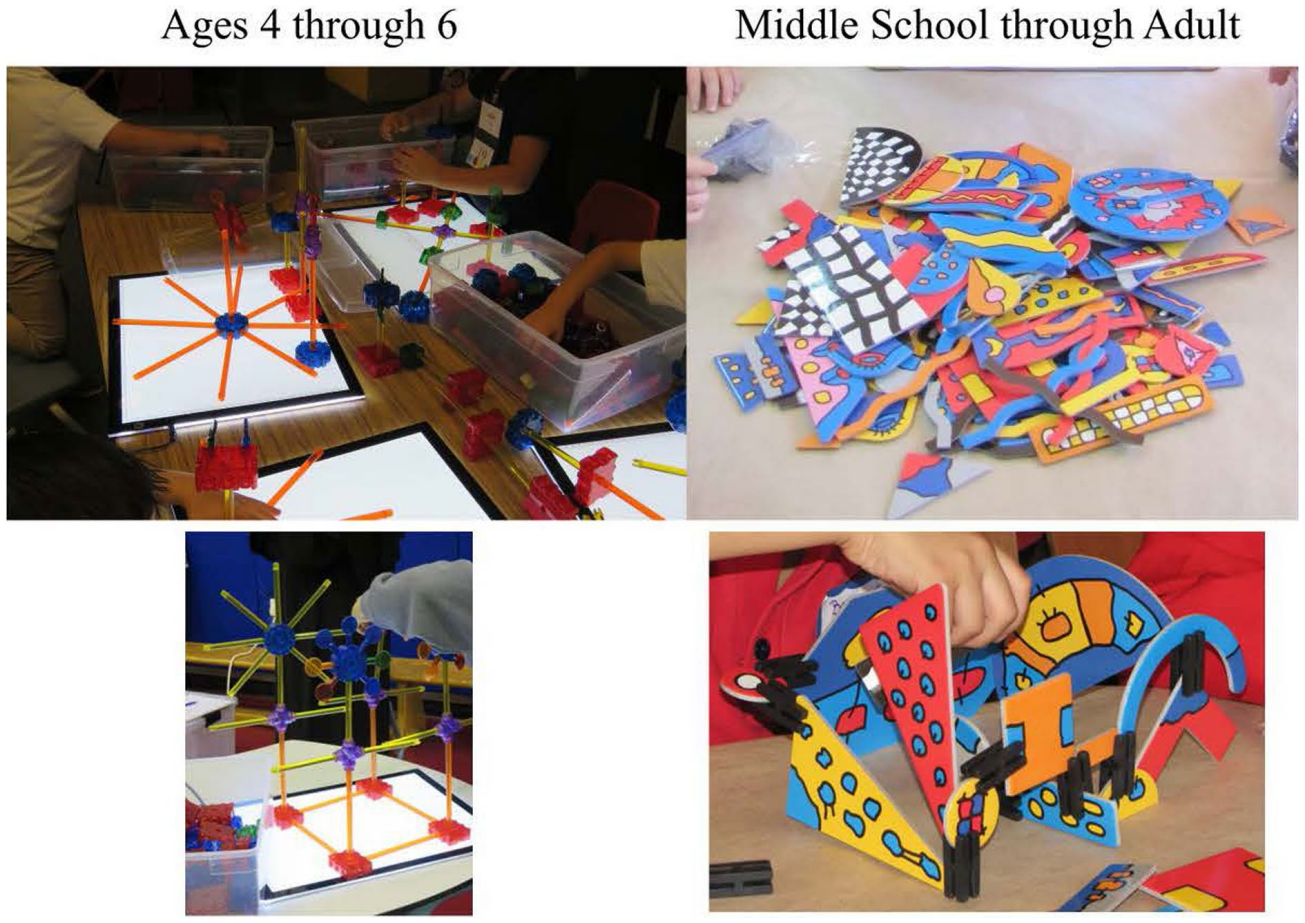
Materials for visual/spatial assessments for all levels.

Choosing materials that can be used in a variety of ways that do not suggest certain kinds of products is very important for this assessment. Figure 9 shows examples of materials we have found flexible for a variety of constructions. They are interesting, engaging, and developmentally appropriate. For young children, the light table is different and adds an interesting dimension, but is not essential. The pieces can be connected in a variety of ways that are easy for most children. The materials for elementary school through adult are colorful and have abstract designs that can be used to enhance the visual appeal of a product. Each participant is given a bag of connectors that enable him/her to make three-dimensional constructions.

#### Open-ended tasks and questions to stimulate creativity

For all ages and levels, the task is the same: “*Make anything you want to make*.” After the participant has completed a construction or the time limit is reached, interview each one: “*Tell me about what you made*.” Be sure *not* to ask “*What is it?*” This is a closed question and is in some ways an insult because it shows you cannot tell what it is, and also implies it has to be something. If participants are asked to tell *about* what they made, they are invited to talk about their process as well as the product.

#### Observable behaviors based on research that are indicators of visual/spatial ability

- Includes many pieces.
- Includes many types of pieces.
- Observes what he/she has made and revises it.
- Construction shows clear resemblance to what he/she says it is.
- Construction implies movement.
- Construction moves.
- Construction shows a relationship to the environment or other items.
- Construction is unique.
- Construction is detailed.
- Construction has abstract elements.
- Construction is symmetrical.
- Construction is asymmetrical with attention to detail and design.

#### Sample visual/spatial products that exhibit creativity

**Figure 10:**
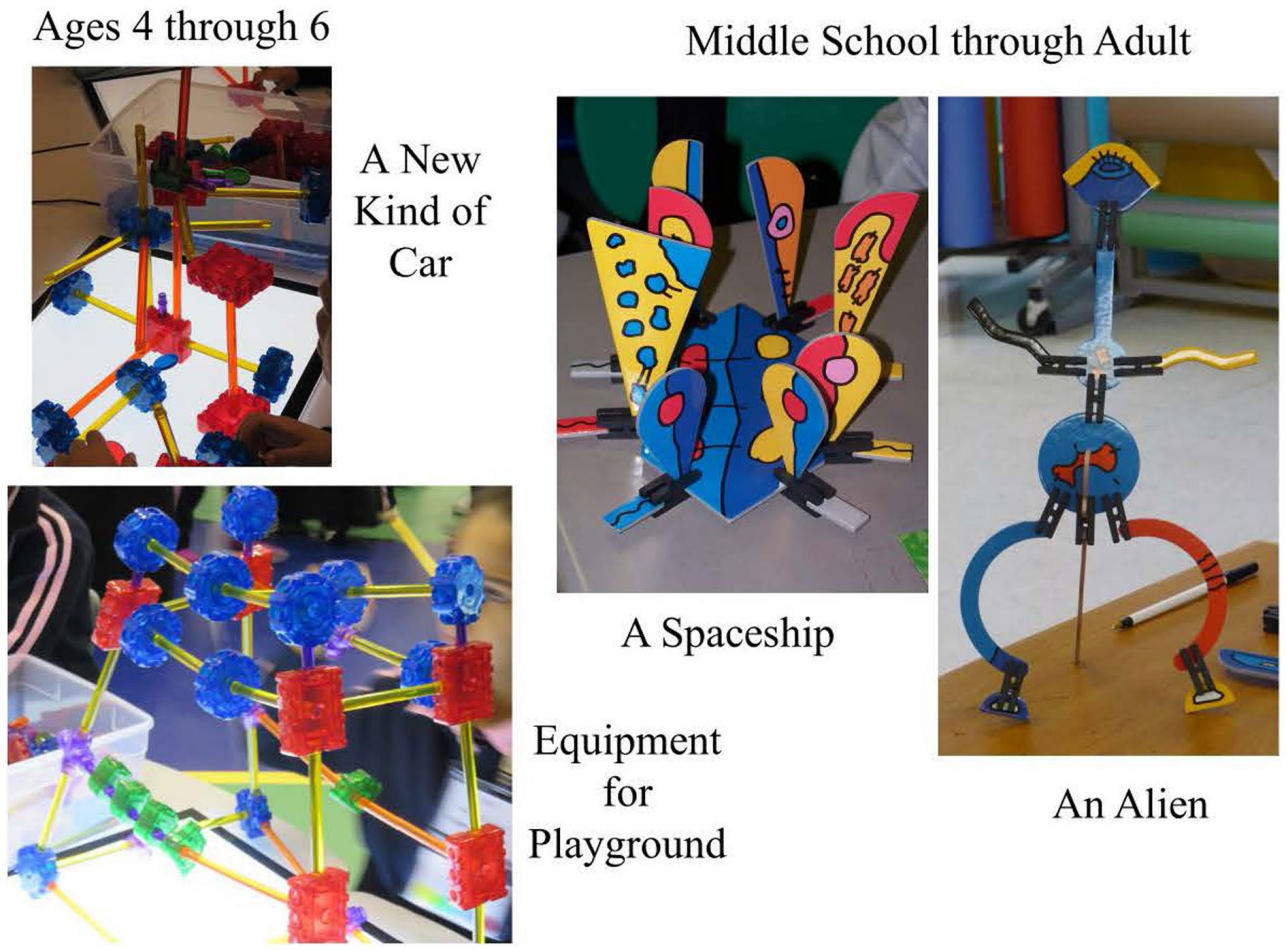
Examples of creative visual/spatial constructions.

When observing, processes used by participants are observed. Products resulting from that process also can be evaluated. Both are important. Figure 10 has examples of visual/spatial products that exhibit creativity.

### Motivation: all domains, tasks, and age levels

During development, field-testing, revision, and validation, certain behaviors have been identified by observers and researchers in all domains and tasks. They can be observed at all levels of education and ages of participants. Observers also are encouraged to write other behaviors they see that they believe indicate motivation. These are examined and added to checklists if they are observable and not inferences.

#### Observable behaviors based on research that are indicators of motivation

- Shows involvement in task (focuses on own task rather than that of others, not easily distracted).
- Works continuously.
- Persists on tasks or activities.
- Increases in motivation or enjoyment as problems increase in open-endedness.
- Follows through to completion.
- Does not want to quit even when others have finished.
- Shows non-verbal enjoyment of task or activity (smiling, laughing, playing).
- Verbalizes enjoyment of task.
- Other (specify) _____________.

## Using the assessments and checklists of behaviors

### Minimizing cultural and economic biases

In the beginning, the DISCOVER assessments were designed as a way to find the most talented and gifted students from all cultures and economic levels. IQ tests, the most accepted method of identification, have been, for many years, biased against students from certain cultures and from low-income backgrounds (c.f., Maker, 1996, 2020b; Miller, 2004; National Science Board, 2010; Rojas, 2015). The perspective of the DISCOVER research teams also included a belief that solving a variety of types of problems, including those without right or acceptable answers is more important than simply knowing the right answer or the most acceptable method for solving a problem (Bahar, 2013; Jonassen, 2000; Maker, 1993, 2005). Research on the assessments showed they were not biased against any groups (Maker, 1996, 2005; Sarouphim, 1999, 2000, 2001, 2002, 2004; Sarouphim and Maker, 2010).

### Responding to the needs of the 21st century

The original perspective is even more important in the 21st century context (Bahar et al., 2024; Maker, 2021; Maker et al., 2024; Maker et al., 2023b; Maker and Bahar, 2024). Information is easily available, critical thinking is needed to evaluate the validity of the information, and creative thinking is essential for using the information in new and innovative ways. Chief Executive Officers (CEOs) and other leaders have identified the 21st century skill of creativity as the most important quality for their top managers, along with critical thinking, collaboration, and communication (Audretsch and Thurik, 2001; Berman and Korsten, 2010; Hiranuma and Kang, 2022; Thornhill-Miller et al., 2023; Thurik et al., 2013). All these skills can be assessed using the DISCOVER performance-and play-based assessments.

### Settings, tasks, checklists, and talent profiles

Preparing for assessments has included identifying a setting such as a classroom or all-purpose room with tables and chairs that can be moved or a small room in which individuals can be assessed. When using the checklists in conjunction with learning centers, a large room or classroom is appropriate. If using the checklists as part of an assessment, students are placed in small groups of 4 to 5, and an observer is assigned to each group. In some cases, the observers rotate from table to table, taking their materials with them. This practice eliminates potential bias in favor of students who performed well in the previous tasks. In other cases, children move from table to table because observers have specialized in a particular set of tasks and have materials that are not moved easily from table to table. All observers have checklists to use during the assessment. They also have cameras or tablets for taking photos and recorders or tablets for recording student responses.

Instead of using the assessments to *identify* individuals who have the highest levels of ability, their best use is to *describe* an individual’s level of talent in varied domains (Maker et al., 2024; Maker and Bahar, 2024). Talent profiles are created for each individual and accompanied by recommendations for nurturing talents that are practical for educators, caregivers, parents, and the individuals themselves (Maker et al., 2023a; Maker et al., 2024; Pease et al., 2020). Supplementary Figure 1 shows a talent profile of a young child and a high school student who participated in Science, Technology, Engineering, and Mathematics (STEM) assessments.

## Conclusion

*Unlocking* and *nurturing* creative problem solving happens when individuals are offered opportunities and encouraged to participate in interesting, engaging, and challenging experiences in which they are presented with open-ended tasks similar to the ones described in this article. One practical, well-tested, and effective way to do this is to set up centers for the different abilities and give students time to explore. In classrooms at preschool and elementary levels, the DISCOVER team worked with teachers to set up centers and give students at least an hour a day to explore and engage in the problem-solving experiences (Maker et al., 2006, 2008). In middle and high school, teachers devoted at least one period a week to free exploration in the areas related to the content they were teaching. For instance, in a physics class, students were encouraged to create machines and vehicles that demonstrated the principles they were learning. In Thailand (Anuruthwong, 2002), school and community centers have been designed. In the centers in schools, teachers schedule time for their classes to participate in the exploring center; and in the centers in communities, both in Thailand and other countries, parents, teachers, and children can participate freely (Maker et al., 2015). Supplementary Figure 2 shows examples of some centers.

Another way to unlock and nurture creative problem solving is through model-building (Maker, 2013). An inexpensive method connected to the curriculum is to give students in small groups the task of creating models related to what they are learning. For example, when studying natural science, they can create models of ecosystems or models of natural changes in the earth; when studying history, they can create models showing a particular period of time, and in math, they can create models of a particular mathematical operation or principle. Junk and other unused materials can be substituted for expensive materials, and their use may require even higher levels of creativity than using materials that can be purchased. Making models of abstract ideas such as equity, beauty, love, and timelessness can be even more challenging than making models of something tangible.

In many classrooms during students’ problem-solving experiences using the Real Engagement in Active Problem Solving (REAPS; Bahar et al., 2021; Maker and Wearne, 2020; Maker and Pease, 2021; Maker et al., 2021; Maker et al., 2022) teaching model and following the Thinking Actively in a Social Context problem solving method (TASC; Wallace et al., 2012), students created models of their solutions and methods for communication of solutions to audiences. To enhance the experience and involve other talents, they can add sounds, verbal descriptions, and other qualities to enrich their presentations (Maker, 2013). Supplementary Figure 3 shows some examples of models made by students.

*Assessing* creative problem solving can be accomplished in both formal and informal settings. The tasks described in this article have been developed for use in formal settings as a way to describe the level of talent development of individuals of all ages in different talent areas. Tasks similar to these can be incorporated into the centers and model-building experiences described in this section. The behaviors are the same regardless of whether the setting is formal or informal. An important caveat to remember is that if people will be assessed using the materials and tasks described, the materials should not be available in the centers or for model-building. They will not be novel, so if some individuals have had experiences with the materials, they may not be as interested or may tend to make something they have made previously. People with prior exposure also might have an advantage over others who have not experienced the materials. Teachers can observe students as they participate in centers using checklists of behaviors, and they can ask students to submit photos, audio recordings, or other records of their products. To document the evolution of talents, portfolios of products, teacher and student descriptions of the process, and behavior checklists completed by observers and the participants themselves are effective and very important. Hopefully, the information and resources in this article will be helpful for everyone, especially the children, youth, and adults who participate.
