# Supplementary material for: Observation: unlocking, assessing, and nurturing creative problem solving
Source: Front Psychol. 2025 Jun 2;16:1540501. doi: 10.3389/fpsyg.2025.1540501 (PMC12168160; doi:10.3389/fpsyg.2025.1540501)
Supplement: Supplementary file 1 [file Supplementary_file_1.docx]

**References for Additional Resources**

**Development and Field-testing of Assessments and Behavior Checklists**

Alfaiz, F., Pease, R., & Maker, C. J. (2020). Culturally responsive assessment of physical science

skills and abilities: Development, field testing, implementation, and results. *Journal of Advanced Academics*. *31*(3) 298–328. <https://journals.sagepub.com/doi/10.1177/1932202X20920572>

Bahar, K., & Maker, C. J. (2020). Culturally responsive assessments of mathematical skills and

abilities: Development, field testing, and implementation. *Journal of Advanced Academics*. *31*(3) 211–233 <https://journals.sagepub.com/doi/10.1177/1932202X20906130>

Maker, C. J. (1994). Authentic assessment of problem solving and giftedness in secondary school students. *Journal of Secondary Gifted Education, 2*(1), 19-29.

<https://eric.ed.gov/?q=learning+and+multiple+and+representations&pg=17914&id=EJ497602>

Maker, C. J. (1996). Identification of gifted minority students: A national problem, needed

changes, and a promising solution. *Gifted Child Quarterly, 40*(1), 41-50. <https://doi.org/10.1177/001698629604000106>

Maker, C. J. (2001). DISCOVER: Assessing and developing problem solving. *Gifted Education*

*International*, *15*(3), 232-251. <https://doi.org/10.1177/026142940101500303>

Maker, C. J. (2005). *The DISCOVER Project: Improving Assessment and Curriculum for Diverse Gifted Learners*. Senior Scholars Series Monograph. Storrs, CT: National Research Center on the Gifted and Talented.

Maker, C. J. (2020a). Culturally responsive assessments of spatial analytical skills and abilities: Development, field testing, and implementation. *Journal of Advanced Academics*. *31*(3) 234–253. <https://doi.org/10.1177/1932202X20910697>

Rogers, J. A. (1998). Refocusing the lens: Using observation to assess and identify gifted learners. *Gifted Education International*, 12(3), 129-144.

<https://doi.org/10.1177/026142949801200302>

Tan, S. & Maker, C. J. (2020). Assessing creative problem solving ability in mathematics: The DISCOVER Mathematics Assessment, *Gifted and Talented International*, *35*(1), 58-71.

<https://doi.org/10.1080/15332276.2020.1793702>

Zimmerman, R. H., Maker, C. J., & Alfaiz, F. S. (2020). Culturally responsive assessment of life science skills and abilities: Development, field testing, implementation, and results. *Journal of Advanced Academics*. 31(3), 329–366.

<https://doi.org/10.1177/1932202X20923981>

**Studies of Validity and Reliability of Assessments**

Alhusaini, A. A., & Maker, C. J. (2018). Who is gifted? The stability of scores on the DISCOVER assessment and the Raven’s progressive matrices in Diné gifted children. *Turkish Journal of Giftedness and Education, 8,* 2, 114-142.

Bahar, K. A., Can, I., & Maker, C. J. (2024). What does it take to be original? A domain-specific exploration of mathematical problem solving. *Thinking Skills and Creativity,* TSC_101592.

Erdimez, O. & Maker, C. J. (2015). *The Predictive Validity of the DISCOVER Performance-Based Assessment to Identify the Academic Achievement of Diné Students*. Unpublished manuscript. Department of Disability and Psychoeducational Studies, University of Arizona, Tucson, Arizona, USA.

Maker, C. J. (2020b). Identifying exceptional talent in science, technology, engineering, and mathematics: Increasing diversity and assessing creative problem-solving. *Journal of Advanced Academics*. *31*(3), 161–210. <https://doi.org/10.1177/1932202X20918203>

Nielson, A. B. (1994). Traditional identification: Elitist, racist, sexist? New evidence. *CAG Communicator: The Journal of the California Association for the Gifted, 25*(3), 18-19, 26-31. <https://eric.ed.gov/?id=ED378782>

Reid, C., Udall, A., Romanoff, B. & Algozzine, B. (1999). Comparison of traditional and problem solving assessment criteria. *Gifted child Quarterly*, *43*(4), 244-251.

<https://doi.org/10.1177/001698629904300404>

Romanoff, B.S., Algozzine, B., & Nielson, A.B. (2009). Achievement of African American and Caucasian students referred and placed or not placed in gifted programs. *Journal for the Education of the Gifted, 33*(2), 156-175. <https://doi.org/10.1177/016235320903300202>

Sak, U., & Maker, C. J. (2003). The long-term predictive validity of a performance-based assessment used to identify gifted CLD students. In *Proceedings of the 15th Biennial World Conference of the World Council for Gifted and Talented Students*.

Sak, U. & Maker, C. J. (2005). Divergence and convergence of mental forces of children in open and closed mathematical problems. *International Education Journal,6* (2), 252- 260. <https://files.eric.ed.gov/fulltext/EJ854978.pdf>

Sak, U. & Maker, C. J. (2006). Developmental variation in children’s creative mathematical thinking as a function of schooling, age, and knowledge. *Creativity Research Journal, 18*(3), 279-291. <https://doi.org/10.1207/s15326934crj1803_5>

Sarouphim, K. A. (1999). DISCOVER: A promising alternative assessment for the identification of gifted minorities. *Gifted Child Quarterly, 43*(4),

<https://doi.org/10.1177/001698629904300403>

Sarouphim, K. A. (2000). Internal structure of DISCOVER: A performance-based assessment. *Journal for the Education of the Gifted. 23*(3), 314-317. <http://eric.ed.gov/?id=EJ609790>

Sarouphim, K. M. (2001). DISCOVER: Concurrent validity, gender differences, and identification of minority students. *Gifted Child Quarterly*, *45*, 130–138. <https://doi.org/10.1177/001698620104500206>

Sarouphim, K. M. (2002). DISCOVER in high school: Identifying gifted Hispanic and Native American students. *The Journal of Secondary Gifted Education, 14*, 30–38. <https://doi.org/10.4219/jsge-2002-385>

Sarouphim, K. M. (2004). DISCOVER in middle school: Identifying gifted minority students. *Prufrock Journal*, *15*(2), 61-69. <https://doi.org/10.4219/jsge-2004-446>

**Implementation and Research on Assessments in Different Countries**

Lori, A.A. (1998). Storytelling and personal traits: Investigating the relationship between children’s storytelling ability and their interpersonal and intrapersonal traits. *Gifted Education International. 13*, 175-184). <https://doi.org/10.1177/026142949801300210>

Sarouphim, K. & Maker, C.J. (2010). Ethnic and gender differences in identifying gifted students: A multicultural analysis. *International Education*, *39* (2), 42-56. <https://trace.tennessee.edu/internationaleducation/vol39/iss2/4>

Sarouphim, K. M. (2009). The use of a performance assessment for identifying gifted Lebanese students: Is DISCOVER effective? *Journal for the Education of the Gifted*. 33(2), 275-295. <https://doi.org/10.1177/016235320903300206>

**Unlocking and Nurturing Creative Problem Solving in Classrooms and Communities**

Alhusaini, A. (2018). Using the TASC Model to Develop Gifted Students’ Creativity: Analytical Review. *Journal for the Education of Gifted Young Scientists, 6*(3), 10-29. <http://dx.doi.org/10.17478/JEGYS.2018.78>

Anuruthwong, U. (2002). Exploring center: Ignition key to children’s potential and thinking.

In U. Anuruthwong, S. Hiranburana, & Piboonchoil, C. *Igniting Children’s Potentials and Creativity: Proceedings of the 7th Asia-Pacific Conference on Giftedness,* 92-93. Center for the Gifted and Talented, Srinakharinwirot University, Bangkok, Thailand.

Bahar, A. K., Maker, C. J., & Scherbakova, A. (2021). The role of teachers’ implementation of the Real Engagement in Active Problem Solving (REAPS) model in developing creative problem solving in mathematics. *Australasian Journal of Gifted Education, 30*(2), 26–39. <https://search.informit.org/doi/10.3316/informit.134990209201977>

Gallagher, S. A., & Gallagher, J. J. (2013). Using Problem-based Learning to explore unseen academic potential. *Interdisciplinary Journal of Problem-based Learning, 7*(1), 111–131. <https://doi.org/10.7771/1541-5015.1322>

Gallagher, S. A. (2015). The role of problem based learning in developing creative expertise.

*Asia-Pacific Educational Review, 16*, 225–235. <https://dx.doi.org/10.1007/s12564-015-9367-8>

Gomez-Arizaga, M., Bahar, K. A., Maker, C. J., Zimmerman, R. H., & Pease, R. (2016). How does science learning occur in the classroom? Students’ perceptions of science instruction during implementation of the REAPS model. *Eurasian Journal of Mathematics and Science Education, 12*(2), 1-24. <https://doi.org/10.12973/eurasia.2016.1209a>

Jo, S. M. & Maker, C. J. (2011). The effect of the DISCOVER curriculum model on mathematical knowledge and creativity. *Asia Pacific Journal of Gifted and Talented Education, 3*(1)*,* 1-17*.*

Maker, C. J., Rogers, J. A., Nielson, A. B., & Bauerle, P. A. (1996). Multiple intelligences, problem solving, and diversity in the general classroom. *Journal for the Education of*

*the Gifted*. *19*(4), 437-460. https://doi.org/10.1177/016235329601900404

Maker, C. J., Muammar, O., Serino, L., Kuang, C. C., Mohamed, A., & Sak, U. (2006). The DISCOVER curriculum model: Nurturing and enhancing creativity in all children. *Korean Educational Development Institute (KEDI) Journal of Educational Policy, 3*(2), 99-121*.* [https://www.semanticscholar.org/paper/The-DISCOVER-curriculummodel%3A- Nurturing-and-in-all-](https://www.semanticscholar.org/paper/The-DISCOVER-curriculummodel%3A-%20Nurturing-and-in-all-) Corpus ID: 148091182

Maker, C. J. Muammar, O., & Jo, S. M. (2008). Development of Creativity: The influence of

traditional and non-traditional pedagogy. *Learning and Individual Differences*. *18*, 402-417. <https://sci-hub.se/10.1016/j.lindif.2008.03.003>

Maker, C. J. & Pease, R. (2008). DISCOVER and TASC in a summer program for gifted students. *Gifted Education International*. *24*(2/3), 323-328. <https://doi.org/10.1177/026142940802400325>

Maker, C. J., Alhusaini, A. A., Pease, R., Zimmerman, R. H., & Alamiri, F. Y. (2015). Developing creativity, talents, and interests across the lifespan: Centers for Creativity and Innovation. *Turkish* *Journal of Giftedness and Education, 5*(2), 83-109.

Maker, C. J. & Wearne, M. (2020). Engaging gifted students in solving real problems creatively: Implementing the Real Engagement in Active Problem Solving (REAPS) teaching/learning model in Australasian and Pacific Rim contexts. In S. R. Smith (Ed.). *International Handbook of* *Giftedness & Talent Development in the Asia-Pacific,* Singapore: Springer International Handbooks of Education. <https://doi.org/10.1007/978-981-13-3021-6_40-1>

Maker, C. J. (2013). Model-building: A practical way to develop creativity in gifted students in

regular classroom settings. *Inspire: The Gifted Education Magazine for Educators. 9,* 10-

14 (English); 15-19 (Chinese).

Maker, C. J., Bahar, K., Alfaiz, F. S., & Pease, R. (2022). Developing and assessing creative

scientific talent that is transformational through Real Engagement in Active Problem Solving (REAPS). *Australasian Journal of Gifted Education, 31*(1), 5–21. <https://search.informit.org/doi/10.3316/informit.376827543162155>

Maker, C. J., Bahar, A. K., Pease, R, & Alfaiz, F. A. (2023). DISCOVERing and nurturing creative problem solving in young children: An exploratory study. *Journal of Creativity, 33*, 1-10. <https://doi.org/10.1016/j.yjoc.2023.100053>

Pease, R., Vuke, M., Maker, C. J., & Muammar, O. M. (2020). A practical guide for implementing the STEM assessment results in classrooms: Using strength-based reports and real engagement in active problem solving. *Journal of Advanced Academics*. *31*(3) 367–406. DOI: 10.1177/1932202X20911643

Riley, T., Webber, M., & Sylva, K. (2017). Real engagement in active problem solving for Māori

boys: A case study in a New Zealand secondary school. *Gifted and Talented International, 32*(2), 75–86. <https://doi.org/10.1080/15332276.2018.1522240>

Wallace, B., Bernardelli, A., Molyneux, C., Farrell, C., & Eriksson, G. (2012). TASC: Thinking

Actively in a Social Context. A universal problem solving process: A powerful tool to promote differentiated learning experiences. *Gifted Education International, 28*(1) 58– 83. https://doi.org 10.1177/0261429411427645

Wallace, B., Maker, C. J., Cave, D., & Chandler, S. (2004). *Thinking skills and problem-solving:*

*An inclusive approach.* London: David Fulton Publishers.

Webber, M., Riley, T., Sylva, K., & Scobie-Jennings, E. (2018). The Ruamano project: Raising

expectations, realising community aspirations and recognising gifted potential in Māori

boys. *The Australian Journal of Indigenous Education*. Advance online publication. <https://doi.org/10.1017/jie.2018.16>

Wu, I-C., Pease, R., & Maker, C. J. (2021). General education teachers’ perceptions of the Real

Engagement in Active Problem Solving (REAPS) model. *Australasian Journal of Gifted Education, 30*(2), 64-79. https://doi.org/ 10.21505/ajge.2021.0015

https://doi.org/10.1080/02783193.2023.2285045

**Recommendations for Use of these Ideas in the 21^st^ Century Context**

Maker, C. J. (2021). Exceptional talent in the 21st century context: Conceptual framework,

definition, assessment, and development. *Gifted Education International, 37*(2) 158–198. <https://doi.org/10.1177/0261429421995188>

Maker, C. J., Pease, R. & Zimmerman, R.H. (2023). Identifying and cultivating innovators and

increasing diversity in Science, Technology, Engineering, and Mathematics (STEM): A Needed Paradigm Shift. *Roeper Review,45*(3), 161-177. <https://doi.org/10.1080/02783193.2023.2212362>

Maker, C. J. (2024a, April). Changing our thinking and practices to fit the 21st Century context.

*WorldTalentWeb Newsletter. 29,* 8-15*.* https://wgc.ae/newsletter

Maker, C. J. & Bahar, A. K. (2024). Talent development in inclusive classrooms: An analysis of

student growth. *The* *Australasian Journal of Gifted Education,33*(2), 28-56. DOI: 10.21505/ajge.2024.0013

Maker, C. J. & Zimmerman, R.H. (2024). Developing Diverse Talents in Mainstream Settings: A 21st Century Alternative to Identifying Gifted Students for Special Classes, Special Programs, and Special Schools. Manuscript submitted for publication.

Sternberg, R. J. (2020). Transformational giftedness: Rethinking our paradigm for gifted education. *Roeper Review, 42*(4), 230-240. <https://doi.org/10.1080/02783193.2020.181526>
