# Supplementary figures and images for: Observation: unlocking, assessing, and nurturing creative problem solving
Source: Front Psychol. 2025 Jun 2;16:1540501. doi: 10.3389/fpsyg.2025.1540501 (PMC12168160; doi:10.3389/fpsyg.2025.1540501)

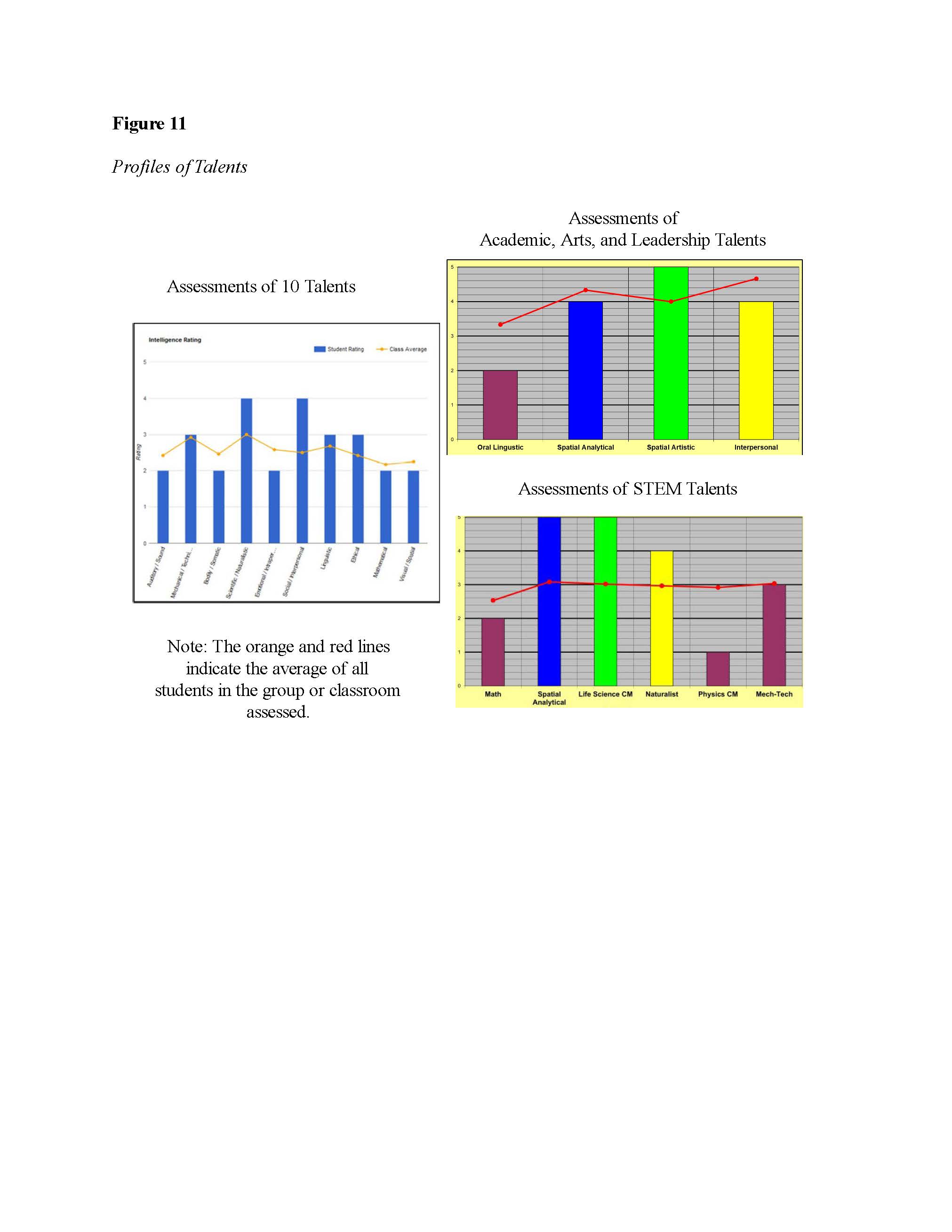

Supplement: Supplementary file 2 [file Image_1.JPEG]

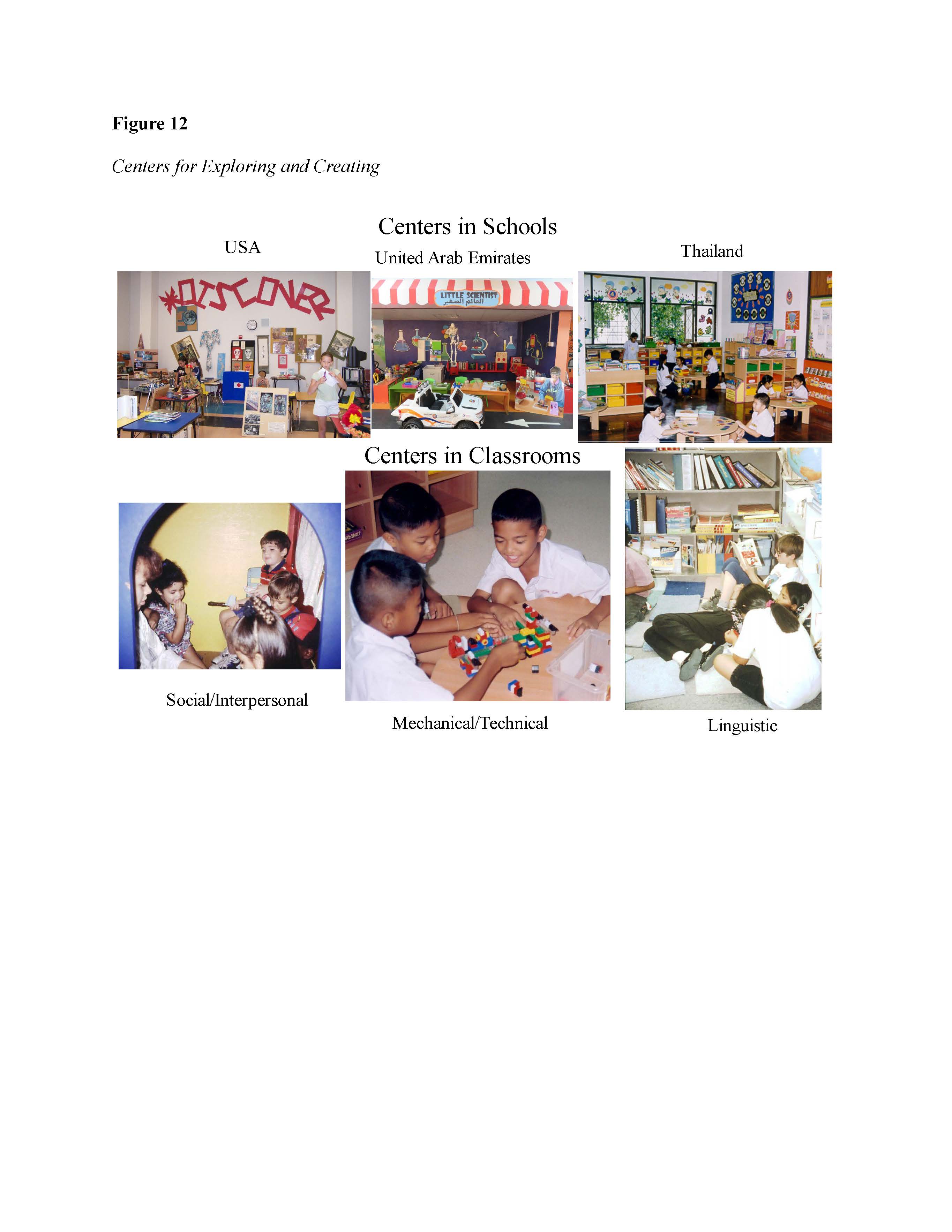

Supplement: Supplementary file 3 [file Image_2.JPEG]

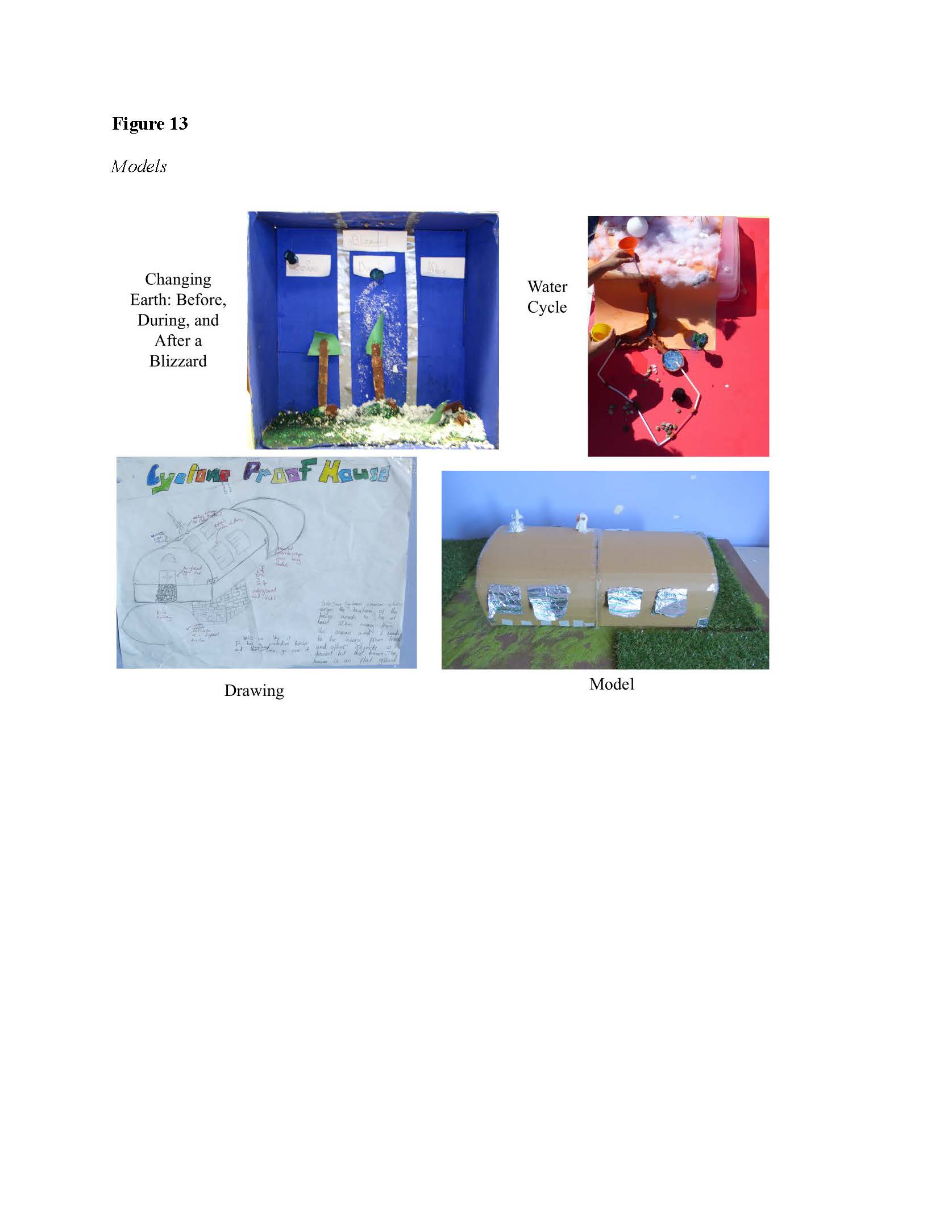

Supplement: Supplementary file 4 [file Image_3.JPEG]
